# Low Physical Activity in Patients with Complicated Type 2 Diabetes Mellitus Is Associated with Low Muscle Mass and Low Protein Intake

**DOI:** 10.3390/jcm9103104

**Published:** 2020-09-25

**Authors:** Ilse J. M. Hagedoorn, Niala den Braber, Milou M. Oosterwijk, Christina M. Gant, Gerjan Navis, Miriam M. R. Vollenbroek-Hutten, Bert-Jan F. van Beijnum, Stephan J. L. Bakker, Gozewijn D. Laverman

**Affiliations:** 1 Division of Nephrology, Department of Internal Medicine, Ziekenhuisgroep Twente, 7609 PP Almelo, The Netherlands; n.braber@zgt.nl (N.d.B); Mi.oosterwijk@zgt.nl (M.M.O.); m.vollenbroek@zgt.nl (M.M.R.V.-H.); g.laverman@zgt.nl (G.D.L.); 2 Faculty of Electrical Engineering, Mathematics and Computer Science, University of Twente, 7522 NB Enschede, The Netherlands; b.j.f.vanbeijnum@utwente.nl; 3 Division of Nephrology, Department of Internal Medicine, University of Groningen, University Medical Center Groningen, 9713 GZ Groningen, The Netherlands; cm.gant@meandermc.nl (C.M.G.); g.j.navis@umcg.nl (G.N); s.j.l.bakker@umcg.nl (S.J.L.B.); 4 Department of Internal Medicine, Meander Medisch Centrum, 3813 TZ Amersfoort, The Netherlands

**Keywords:** type 2 diabetes, physical activity, muscle mass, protein intake, accelerometer

## Abstract

Objective: In order to promote physical activity (PA) in patients with complicated type 2 diabetes, a better understanding of daily movement is required. We (1) objectively assessed PA in patients with type 2 diabetes, and (2) studied the association between muscle mass, dietary protein intake, and PA. Methods*:* We performed cross-sectional analyses in all patients included in the Diabetes and Lifestyle Cohort Twente (DIALECT) between November 2016 and November 2018. Patients were divided into four groups: <5000, 5000–6999, 7000–9999, ≥ 10,000 steps/day. We studied the association between muscle mass (24 h urinary creatinine excretion rate, CER) and protein intake (by Maroni formula), and the main outcome variable PA (steps/day, Fitbit Flex device) using multivariate linear regression analyses. Results: In the 217 included patients, the median steps/day were 6118 (4115–8638). Of these patients, 48 patients (22%) took 7000–9999 steps/day, 37 patients (17%) took ≥ 10,000 steps/day, and 78 patients (36%) took <5000 steps/day. Patients with <5000 steps/day had, in comparison to patients who took ≥10,000 steps/day, a higher body mass index (BMI) (33 ± 6 vs. 30 ± 5 kg/m^2^, *p* = 0.009), lower CER (11.7 ± 4.8 vs. 14.8 ± 3.8 mmol/24 h, *p* = 0.001), and lower protein intake (0.84 ± 0.29 vs. 1.08 ± 0.22 g/kg/day, *p* < 0.001). Both creatinine excretion (β = 0.26, *p* < 0.001) and dietary protein intake (β = 0.31, *p* < 0.001) were strongly associated with PA, which remained unchanged after adjustment for potential confounders. Conclusions: Prevalent insufficient protein intake and low muscle mass co-exist in obese patients with low physical activity. Dedicated intervention studies are needed to study the role of sufficient protein intake and physical activity in increasing or maintaining muscle mass in patients with type 2 diabetes.

## 1. Introduction

Type 2 diabetes is a predominately lifestyle-related disease and has become one of the major global public health concerns, with highest prevalence in older adults [1]. Sufficient physical activity (PA) is a main focus of treatment, in addition to improving diet quality. There are two different aspects of PA: aerobic training and resistance exercise. While guidelines first mainly recommended moderate to vigorous PA, contemporary public health guidelines state that ‘some physical activity is better than none’ by suggest reducing the time spent in sedentary behaviour [2]. Total steps per day is a good indicator of the overall volume of physical activity [3]. 

However, the vast majority of patients with type 2 diabetes do not adhere to the American Diabetes Association (ADA) guidelines of >150 min per week of moderate to vigorous PA, which is comparable with 7000 steps per day [3,4,5]. Traditionally, a goal of 10,000 steps per day has been advocated by popular media, although this goal is under debate in scientific literature [6,7]. In order to promote PA and reduce sedentary behaviour, a better understanding of total daily movement is required, especially in patients with complicated type 2 diabetes. 

In regard to PA, sufficient muscle mass is mandatory to perform PA, and conversely, PA promotes an increase in muscle mass. Compared with non-diabetic subjects, patients with type 2 diabetes show decreased muscle strength and mass [8,9]. In type 2 diabetes, reduced muscle mass and muscle function, defined as sarcopenia, have been implicated both as a cause and as a consequence of increased insulin resistance [8,9,10]. Furthermore, it is known that low muscle mass in obese individuals is associated with frailty, disability, and increased morbidity and mortality [11].

However, dietary counselling (such as is performed in the geriatric population) consists mainly of caloric restriction, and not the preservation of muscle mass. Adequate protein intake is an important requirement for sustaining, and especially increasing, muscle mass, which has been confirmed by several observational and intervention studies [12,13,14,15,16,17]. Moreover, combining physical exercise with protein intake has a positive synergistic effect on muscle protein synthesis [16,17]. Therefore, adequate protein intake might be a current blind spot in the treatment of type 2 diabetes.

We hypothesize that in patients with complicated type 2 diabetes, low protein intake and low muscle mass are associated with low PA, and the former could be an important actionable item to improve PA. Therefore, here we (1) objectively measure PA (in steps/day) in patients with complicated type 2 diabetes, and (2) investigate the association between protein intake and muscle mass and PA.

## 2. Materials and Methods

### 2.1. Patient Inclusion

This study was performed in the DIAbetes and LifEstyle Cohort Twente (DIALECT), an observational cohort study in patients with complicated type 2 diabetes mellitus, treated in the secondary healthcare level in the outpatient clinic of the Ziekenhuisgroep Twente (ZGT), Almelo and Hengelo, the Netherlands. The study consists of two sub-cohorts: DIALECT-1 and DIALECT-2. The general procedures have been described extensively previously [18]. In DIALECT-2, the data collection at baseline is more extensive, including a one-week PA registration.

The study was performed in accordance with the Helsinki agreement and the guidelines of good clinical practice, has been approved by the local institutional review boards (METC-registration numbers NL57219.044.16 and 1009.68020) and is registered in the Netherlands Trial Register (NTR trial code 5855). Prior to participation, all patients signed an informed consent form. All adult patients with type 2 diabetes treated in the secondary healthcare level in the outpatient clinic of internal medicine in ZGT Hospital were eligible for participation. The patients were treated in the secondary healthcare level because the diabetes care became complex for primary healthcare services (for example, in the presence of complications such as nephropathy or because of a complex insulin schedule). Exclusion criteria were renal replacement therapy, inability to understand the informed consent procedure, and inability to walk. We report here on all patients included in DIALECT-2 between November 2016 and November 2018. 

### 2.2. Data Collection

Participation in DIALECT-2 consisted of at least two hospital visits with one week in between. Information on medical condition and medication was obtained from electronic patient files and verified with the patient during the baseline visit. Smoking habits were collected through questionnaires. Anthropometric measurements, leg length, and presence of diabetic polyneuropathy were obtained from physical examination at baseline. Leg length was measured using a tape measure from the anterior superior iliac spine to the ground. Polyneuropathy was assessed by touch test (Semmes Weinstein monofilament) and vibration (Vibratip) by the on–off method; both tests have been validated as screening methods for polyneuropathy [19]. Polyneuropathy was present if at least one of the two tests was positive. Body composition parameters were determined by Bio impedance using a TANITA device (type BC-418MA, Tokyo, Japan), which calculates segmental body composition, including fat percentage and predicted muscle mass. Blood samples were taken from a single non-fasting venapunction, and patients collected 24 h urine to provide objective data on nutritional intake, including protein intake. We used the 24 h urinary creatinine excretion rate (CER) as a measure of muscle mass [11,20,21]. The estimated daily protein intake (g/kg/day) was calculated using the universally adopted formula of Maroni, ((24 h urea excretion × 0.18) + 15 + 24 h protein excretion)/weight (kg) [22]. Blood pressure was measured in supine position by an automated device for 15 min with one-minute intervals (Dinamap^®^; GE Medical systems, Milwaukee, WI, USA). The mean systolic and diastolic pressure of the last three measurements was used for further analysis. 

### 2.3. Main Outcome: Physical Activity

During 8 consecutive days, daily movement was measured by a triaxial Fitbit accelerometer worn around the wrist on the non-dominant side. The devices used were either a Fitbit Flex (Fitbit Inc., Boston, MA, USA), a Fitbit charge HR (Fitbit, San Francisco, CA, USA), or Fitbit Charge 2 (Fitbit Inc., San Francisco, CA, USA). These Fitbit devices share the same recording mechanisms and record the number of steps taken on a minute-to-minute basis. Patients were asked to adhere to their daily activities as normal and were blinded from the online activity data. Also, the Fitbit screens showed no results. Only during swimming or showering was the Fitbit removed. At visit 2 (day 8), the patients returned the Fitbit and data were transferred to a hospital server for further analysis. Patients were asked to write down information regarding non-wearing time in a lifestyle diary. Valid days were defined as days without significant non-wearing time (i.e., >2 h non-wearing time during waking hours). Patients with more than two days of significant non-wearing time were excluded. To indicate the total daily movement, we used the average of the total steps per day, excluding day 1 and 8 from the average because of non-wearing time. 

### 2.4. Statistical Analysis 

Statistical procedures were performed by using SPSS statistics (IBM Statistics for Windows, Version 23.0, Armonk, NY, USA). Normality of data was determined by visual inspection of histograms. Data were presented as mean ± standard deviation (normal distribution), as median and interquartile range (IQR 25th–75th percentiles, skewed data), or as number and percentage (dichotomous and categorical data). To compare the characteristics of total steps per day, the population was divided into four different groups based on reference values from current literature (i.e., <5000, 5000–6999, 7000–9999, ≥10,000 steps per day) [3]. Differences between the groups were analysed using One-Way ANOVA, Kruskal–Wallis, or Chi-square test when appropriate. A two-sided *p* < 0.05 was considered statistically significant. The estimated daily protein intake was divided into three groups (i.e., <0.8 g/kg/day, 0.8–1.2 g/kg/day, and >1.2 g/kg/day). The recommended dietary protein intake is ≥0.8 g/kg/day [12,23]. 

To investigate whether 24 h CER and protein intake were associated with total steps/day, we performed multivariate linear regression analyses. First, we identified possible confounders using univariate analyses. Model 2, adjusted for age and gender, was the main basis for confounder selection. Parameters with a *p* < 0.15 were considered contenders for the multivariate model. For each group of closely associated variables (for example, body mass index (BMI), waist circumference, and hip circumference as measures of body size), we included the variable with the highest β for the multivariate model. Potential interaction of protein intake and CER with total steps/day was evaluated by inclusion of the product term in the linear regression analysis. We considered a *p* < 0.10 to be statistically significant for the product term. To graphically represent the interaction between protein intake, CER, and total steps per day, we created nine groups based on the tertiles of protein intake and tertiles of muscle mass. Low, medium, and high represent respectively the lowest, middle, and highest tertiles of protein intake and muscle mass. 

## 3. Results

### 3.1. Baseline Characteristics and Total Steps per Day

Of 231 eligible participants, 217 patients were included in the study. The reasons for exclusion were: hardware malfunction (*n* = 6), non-fitting wristband (*n* = 4), patient dropped out during participation (*n* = 2), and patient not able to walk (*n* = 2) (Figure S1). Patient characteristics stratified by total steps per day are shown in Table 1. 

Median total steps per day was 6118 (4115–8638, data not shown). Of the total study population, 85 patients (39%) took ≥7000 steps/day, of whom 37 patients (17%) reached ≥10,000 steps/day (Table 1). 

The mean age of the total study population was 65 ± 12 years, two-thirds were men, and the mean BMI was 32 ± 6 kg/m^2^. Of all patients, 64% used insulin, and the mean HbA1c was 60 ± 13 mmol/mol (7.6% ± 3.3%). The prevalence of micro- (74%) and macrovascular (35%) complications was high. Compliance with wearing of the Fitbit sensor was good; 22 patients reported significant non-wearing time (>2 h/day) at any day during day 2 until day 7, however, no patient had more than two days of non-wearing time (compliance data not shown).

The mean age was highest (69 ± 11 years) in the group of patients with <5000 steps per day (*p* < 0.001). There were no differences in gender between the groups (*p* = 0.99). Patients with <5000 steps/day had the highest BMI (33 ± 6 kg/m^2^, *p* = 0.009) and the highest waist- and hip circumference (waist: 116 ± 14 cm, *p* = 0.001; hip: 115 ± 14 cm, *p* = 0.009). Both measurements of muscle mass (i.e., 24 h CER and percentage of predicted muscle mass (PMM) using bio-impedance) were lowest in patients with 5000 steps/day (*p* = 0.001 and *p* = 0.06, respectively). 

**Figure 1:**
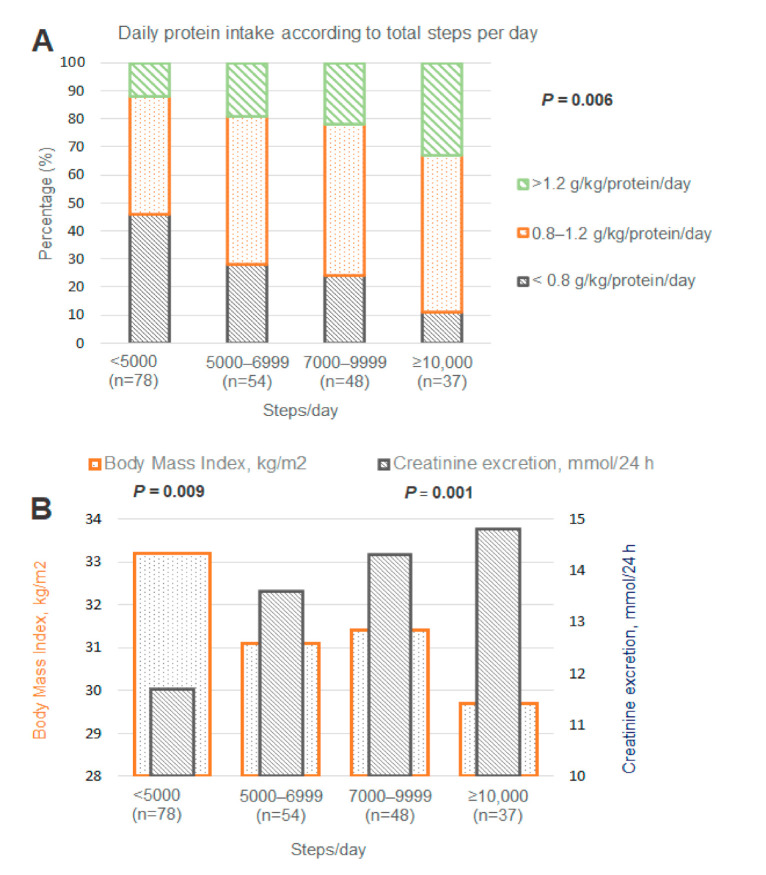
Protein intake, body mass index (BMI), and 24 h urinary creatinine excretion according to total steps per day. Distribution of total protein intake (**A**) and urinary creatinine excretion and body mass index (**B**) in four groups of total steps per day. (**A**) demonstrates that insufficient protein intake is significantly more prevalent in patients with <5000 steps/day. (**B**) shows higher body mass index and lower creatinine excretion in patients with <5000 steps/day, demonstrating a more unfavourable body composition.

No significant differences were observed in HbA1c, insulin use, and years of diabetes between the groups. Patients with <5000 steps per day had the most pack-years of smoking (*p* = 0.005), the lowest diastolic blood pressure (0.02), and the lowest HDL-cholesterol (*p* = 0.03). The prevalence of micro- and macrovascular complications was consistently and progressively lower in each group of increasing number of steps/day, especially for diabetic kidney disease (*p* = 0.004), polyneuropathy (*p* = 0.008), and cerebrovascular disease (*p* = 0.008). Protein intake was also lowest in patients with <5000 steps/day (0.84 g/kg/day, *p* < 0.001, Figure 1). Almost half of all patients with <5000 steps per day had a protein intake <0.8 g/kg/day.

### 3.2. Association between Urinary Creatinine Excretion, Total Protein Intake, and Total Steps per Day

**Figure 2:**
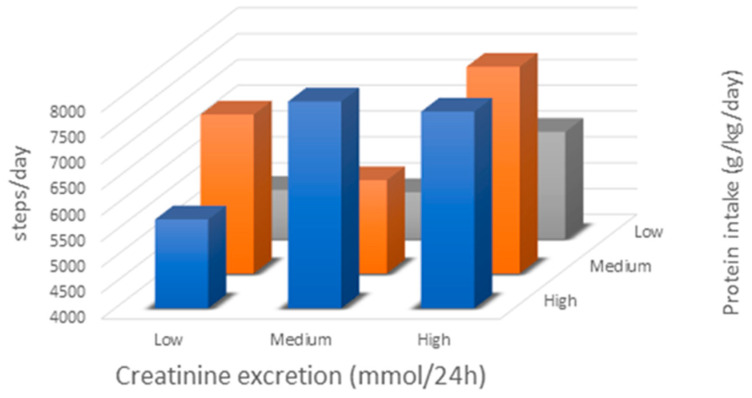
Low, medium, and high represent the lowest, middle, and highest tertiles of protein intake and creatinine excretion. The figure shows the interaction between urinary creatinine excretion (CER) and protein intake on total steps per day. Both high CER and high protein intake were associated with more steps/day, and total steps per day was highest in those with both high CER and high protein intake.

To analyse the association between total steps per day, muscle mass (24 h CER), and daily dietary protein intake, we performed linear regression analyses. Unadjusted, both CER (β = 0.28, *p* = 0.03) and dietary protein intake (β = 0.29, *p* = 0.004) (Model 1) were positively associated with steps/day. When adjusting for possible confounders (Table S1), both for CER and protein intake, the association with total steps/day did not markedly change (Table 2). It should be noted that the predicted variance of both models remained low (0.23 and 0.24, respectively). There was a significant interaction between CER and protein intake on total steps per day, where higher CER combined with higher protein was associated with more steps/day (*p* = 0.096, Figure 2). As there was a very strong correlation between CER and dietary protein intake (*R* = 0.57), both variables could not be inserted simultaneously in the analysis.

## 4. Discussion

We investigated the total daily physical activity (PA) of patients with complicated type 2 diabetes. We found that more than one-third of the study participants had limited activity (less than 5000 steps per day). On the other hand, 39% of participants took ≥7000 steps per day, which has been advocated as the movement target for adults ≥ 65 years and/or patients with chronic diseases [3], demonstrating that sufficient PA in a complicated type 2 diabetes population is indeed a reachable goal.

Our main finding was that low muscle mass was an important determinant of low PA. Additionally, protein intake was significantly and relevantly lower in patients with both low PA and low muscle mass. It is tempting to speculate on a downward spiral of reduced protein intake, lower muscle mass, and reduced PA, against the background of a sedentary lifestyle. The insight that insufficient protein intake is associated with low muscle mass and physical inactivity may provide an important actionable item to improve physical fitness in patients with type 2 diabetes: namely, increase protein intake. 

Low muscle mass is increasingly recognized as an important health concern in patients with chronic disease, diminishing physical fitness and PA. In contrast to previous beliefs, declining muscle mass is not only due to ageing and physical inactivity, but has many other contributing causes, such as mitochondrial dysfunction [11,24,25]. This is especially important in patients with type 2 diabetes, as data suggest skeletal muscle lipid content is associated with systemic insulin resistance [11]. Damage to the skeletal muscles, with pronounced and accelerated decline in muscle quality, has been described as a new complication of diabetic patients attributed to their longer survival [8]. Insulin resistance and oxidative stress are components of the pathophysiological basis of sarcopenia, which would be related to characteristic components of diabetes, such as vascular alterations, chronic inflammation, and lipid infiltration in muscles [8,11]. In regard to our population, 24 h CER in the group with ≤5000 steps per day (11.7 ± 4.8 mmol/24 h) was significantly lower compared to the total study population (13.2 ± 5 mmol/24 h), and also lower when compared to the general Dutch population (13.3 ± 4.1 mmol/24 h, based on data from the Lifelines cohort study) [12]. However, it should be noted that no diagnostic methods or definitive cut-off points exist to identify patients who might benefit from muscle-boosting therapy.

Adequate protein intake is an important requirement for sustaining, and especially increasing, muscle mass, which has been confirmed by several observational and intervention studies [12,13,14,15,16,17]. Moreover, combining physical exercise with protein intake has a positive synergistic effect on muscle protein synthesis [16,17]. 

The recommended dietary allowance (RDA) and the Netherlands Nutrition Centre [12,23] recommend a dietary protein intake of ≥0.8 g/kg/day. However, for elderly adults, the Dutch guideline suggests a higher protein intake (1.2–2.0 g/kg/day) to maintain optimal muscle health [26,27]. We found that almost half of all patients (46%) in the group of <5000 steps per day had a daily protein intake < 0.8 g/kg/day, and only 12% had an intake of >1.2 g/kg/day. To our knowledge, this is the first study in patients with type 2 diabetes that has highlighted the insufficient protein intake of inactive patients with type 2 diabetes. However, BMI and waist circumference were higher in patients with low PA, consistent with altered body composition in inactive patients. This is in line with previous studies in patients with type 2 diabetes [4,28,29]. Low muscle mass and function have strong negative prognostic impacts in obese individuals, which may lead to frailty disability and increased morbidity and mortality [11]. However, awareness of the importance of muscle maintenance in obesity is low among clinicians and scientists [11]. The European Society for Clinical Nutrition and Metabolism (ESPEN) and the European Association for the study of Obesity (EASO) recognize and identify obesity with altered body composition due to low skeletal muscle function and mass as a scientific and clinical priority for researchers and clinicians. ESPEN and EASO therefore call for action in particular regard to optimal nutritional therapy. Generally, the first step in treating obese patients with type 2 diabetes is weight loss interventions by following a caloric restricted diet, which, however, might increase the risk for undesirable decreases in muscle mass. 

To our knowledge, this is the first study to objectively measure daily PA by using steps/day in complicated type 2 diabetes. Most of the previous studies in type 2 diabetes used metabolic equivalent (MET) or counts per minute (CPM) to measure daily movement, which makes it somewhat difficult to compare previous results with our findings [4,24,25,28,29,30,31,32,33,34]. However, in a study population with older patients (≥55 years) with type 2 diabetes, the average total steps per day was similar to our results [34]. In contrast to this previous study, which showed that older women had fewer steps per day, we found no difference in steps/day between genders.

Additionally, we found that the presence of micro- and macrovascular complications was higher in patients with physical inactivity. This is in line with a recent review on diabetic polyneuropathy and nephropathy [35]. Interestingly, diabetic polyneuropathy is associated with lower muscle strength measured by knee extension force [25,32,35], providing an alternative cause of muscle mass decline in addition to reduced dietary protein intake. Additionally, in patients with chronic kidney disease, uremic muscle mass decline has been suggested by a significant inverse association between uremic toxin indoxyl sulphate and skeletal muscle mass [33,35]. Of note, in our study, a third of the patients with ≥10,000 steps per day had polyneuropathy and nephropathy (28% and 33%, respectively), suggesting that sufficient PA is indeed possible in spite of the presence of these complications. However, in contrast to other studies in patients with type 2 diabetes, we found associations between HDL-cholesterol, diastolic blood pressure, macrovascular complications, and physical activity [4,28,29].

Strengths of our study included the objective measurements of daily movement by the Fitbit Flex, a light and simple wristband, well applicable in daily life clinical practice that hardly interferes with daily activities. We chose to present steps/day, which is easily interpretable by clinicians and patients. Another strength of our study was muscle mass estimation by 24 h CER, which is well accepted for estimation of total body skeletal muscle mass, even in patients with advanced renal failure [12,21]. Additionally, we objectively determined protein intake by 24 h urinary urea excretion. In the future, we plan to extend our analysis to also include muscle quality using gait speed, as well as quality of life questionnaires. An important limitation of our study is the cross-sectional design, which allows only research of associations and not causality. Additional prospective studies are warranted to confirm our findings. Another limitation is that one-week record of the Fitbit may not be sufficiently representative of PA, as certain activities, such as swimming, and seasonal variations were not taken into account. However, only 8 patients of the total 217 patients (4%) recorded swimming in their lifestyle diary. Secondly, we had the sampling periods of our population distributed over the seasons. Making these effect negligible.

Our study has important clinical implications. We found clear associations between low protein intake, loss of muscle mass, and low PA in patients with complicated type 2 diabetes. Our study suggests that optimizing protein intake might be a first step to improving physical fitness in patients with type 2 diabetes. As current dietary guidelines focus on reducing overall caloric intake, and carbohydrate intake in particular, adequate protein intake might be an important blind spot in current nutritional management. This has also been advocated in previous studies, which suggest that dietary protein should be prescribed together with physical exercise in order to optimize muscle health [12,16,17,36]. The review by Scot and colleagues also emphasizes that lifestyle modification programs for older adults with type 2 diabetes, particularly for those with sarcopenia, should incorporate progressive resistance training, along with adequate intakes of protein and vitamin D, which may improve both functional and metabolic health and prevent undesirable decreases in muscle mass associated with weight loss intervention [9]. In the future, we want to include data from the Food Frequency Questionnaire (FFQ) in the analyses in order to investigate how intakes of total energy, carbohydrate, fat, and vitamin D may contribute to muscle mass and physical activity. It is important to note that the source of dietary protein (animal or vegetable) should also be taken into account, as we have previously shown that higher vegetable protein intake is associated with lower prevalence of renal function impairment [37]. 

## 5. Conclusions

In conclusion, our study shows that prevalent low protein intake and low muscle mass co-exist in patients with complicated type 2 diabetes with low physical activity. Dedicated intervention studies are needed to study the role of sufficient protein intake and PA in increasing or maintaining muscle mass in patients with type 2 diabetes. 

## Figures and Tables

**Table 1:** Patient characteristics stratified by total steps per day.

Characteristics	*n*	Total Population (*n* = 217)	<5000 steps/day (*n* = 78)	5000–6999 steps/day (*n* = 54)	7000–9999 steps/day (*n* = 48)	≥10,000 steps/day (*n* = 37)	*p*-Value
Age, years	217	65 ± 12	69 ± 11	64 ± 10	62 ± 13 ^a^	60 ± 10 ^a^	<0.001
Gender, men *n* (%)	217	144 (66)	51 (65)	36 (67)	32 (67)	25 (68)	0.99
BMI, kg/m^2^	217	32 ± 6	33 ± 6	31 ± 5	31 ± 5	30 ± 5 ^a^	0.009
Education level, *n* (%) ^b^	185						0.10
Low		65 (35)	31 (44)	16 (36)	11 (28)	7 (23)	
Medium		81 (44)	29 (41)	17 (38)	16 (41)	19 (63)	
High		39 (21)	11 (16)	12 (27)	12 (31)	4 (13)	
Waist circumference, cm	216	112 ± 13	116 ± 14	109 ± 12 ^a^	111 ± 13	107 ± 10 ^a^	0.001
Hip circumference, cm	216	111 ± 13	115 ± 14	109 ± 12	110 ± 11	108 ± 9 ^a^	0.009
Leg length, cm	120	98 ± 7	96 ± 8	97 ± 6	100 ± 8	99 ± 7	0.11
Fat percentage, %	206	33 ± 9	34 ± 8	33 ± 9	32 ± 10	30 ± 8	0.10
Predicted muscle mass, % ^c^	206	64 ± 8	62 ± 8	63 ± 8	65 ± 8	67 ± 8	0.06
Creatinine excretion, mmol/24 h	215	13.2 ± 5	11.7 ± 4.8	13.6 ± 4.3	14.3 ± 5.1 ^a^	14.8 ± 3.8 ^a^	0.001
Systolic blood pressure, mmHg	211	130 ± 15	132 ± 16	127 ± 14	131 ± 16	133 ± 13	0.26
Diastolic blood pressure, mmHg	211	74 ± 9	72 ± 9	76 ± 9	73 ± 8	77 ± 12^a^	0.02
Pulse rate, bpm	211	71 ± 12	71 ± 13	73 ± 11	69 ± 10	70 ± 13	0.44
Diabetes duration, years	217	13 (8–19)	14 (8–20)	14 (8–19)	13 (5–20)	11 (7–18)	0.65
Insulin use, yes *n* (%)	215	138 (64)	56 (73)	32 (59)	25 (52)	25 (70)	0.09
Units of insulin	135	62 (34–101)	66 (40–118)	78 (38–114)	53 (26–105)	50 (35–77)	0.53
Alcohol intake units/month	213	3 (0–25)	0 (0–24)	5 (0–26)	2 (0–11)	8 (0–30)	0.27
Smoking, pack-years	199	8 (0–24)	14 (1–32)	7 (0–28)	1 (0–19) ^a^	1 (0–21) ^a^	0.005
Hba1c, mmol/mol (%)	215	60 ± 13 (7.6 ± 3.3)	60 ± 13 (7.6 ± 3.3)	60 ± 11 (7.6 ± 3.1)	62 ± 14 (7.8 ± 3.4)	59 ± 11 (7.5 ± 3.1)	0.69
Total cholesterol, mmol/L	215	4.2 ± 1.0	4.2 ± 1.1	4.2 ± 1.0	4.4 ± 0.9	4.2 ± 0.8	0.85
HDL-cholesterol, mmol/L	214	1.13 ± 0.3	1.10 ± 0.3	1.16 ± 0.3	1.15 ± 0.4	1.24 ± 0.3^a^	0.03
LDL-cholesterol, mmol/L	200	2.04 ± 0.8	2.0 ± 0.9	2.03 ± 0.9	2.16 ± 0.8	1.97 ± 0.6	0.67
Microvascular complications, *n* (%)	217	160 (74)	66 (85)	37 (69)	27 (56)	16 (43)	<0.001
Diabetic kidney disease, *n* (%)	211	106 (50)	48 (66)	27 (50)	19 (40)	12 (33)	0.004
eGFR < 60 mL/min/1.73 m^2^, *n* (%)	217	70 (32)	38 (59)	17 (32)	10 (21)	5 (14)	<0.001
Micro-albuminuria, *n* (%)	211	83 (39)	40 (55)	20 (37)	14 (29)	9 (25)	0.006
Polyneuropathy, *n* (%)	217	97 (45)	46 (59)	24 (44)	17 (36)	10 (28)	0.008
Retinopathy, *n* (%)	210	41 (20)	18 (24)	10 (19)	6 (13)	7 (19)	0.532
Macrovascular complications, *n* (%)	217	75 (35)	33 (42)	22 (41)	12 (25)	8 (22)	0.05
Peripheral arterial diseases, *n* (%)	217	8 (4)	4 (5)	1 (2)	2 (4)	1 (3)	0.78
Coronary artery diseases, *n* (%)	216	54 (25)	23 (30)	18 (34)	8 (17)	5 (14)	0.06
Cerebrovascular accident or TIA, *n* (%)	217	27 (12)	17 (22)	6 (11)	1 (2)	3 (8)	0.008
Amputation, *n* (%)	217	3 (2)	3 (4)	0 (0)	0 (0)	0 (0)	0.14
Urea excretion, mmol/24 h	206	387 (291–510)	342 (242–448)	402 (274–505)	432 (327–508) ^a^	465 (331–528) ^a^	0.02
Protein intake, g/day	202	88 ± 28	79 ± 27	90 ± 31	93 ± 24 ^a^	96 ± 24 ^a^	0.004
Protein intake, g/kg/day	202	0.95 ± 0.30	0.84 ± 0.29	0.99 ± 0.33^a^	0.99 ± 0.27 ^a^	1.08 ± 0.22 ^a^	<0.001
<0.8 g/kg /day, *n* (%)		64 (31)	35 (46)	15 (28)	10 (24)	4 (11)	0.006
0.8–1.2 g/kg/day, *n* (%)		102 (50)	32 (42)	28 (53)	22 (54)	20 (56)	
>1.2 g/kg/day, *n* (%)		40 (19)	9 (12)	10 (19)	9 (22)	12 (33)	

^a^ Significant difference from <5000 steps/day. ^b^ Education level according to the International Standard Classification of Education (ISCED), as follows: Low: ISCED 1–2; Medium: ISCED 3; High ISCED 4–8: ^c^ Predicted Muscle Mass %: TANITA predicted muscle mass (kg) divided by total body weight (kg). Data presented as mean ± standard deviation, as median and interquartile range (IQR 25th–75th), or in number and (percentage). eGFR: estimated glomerular filtration rate. TIA: transient ischemic attack.

**Table 2:** Multivariate linear regression analyses on the associations between CER, protein intake and total steps/day (dependent variable)

Independent Variables		Total Steps per day (Dependent)			Independent Variables	Total Steps per day (Dependent)		
		Standardized Beta	*p*-Value	*R* ^2^		Standardized Beta	*p-*Value	*R* ^2^
Model 1	CER	0.28	0.003	0.08	Protein intake	0.29	0.004	0.08
Model 2	CER	0.23	0.03	0.10	Protein intake	0.28	0.004	0.13
Model 3	CER	0.23	0.04	0.19	Protein intake	0.18	0.10	0.19
Model 4	CER	0.26	0.04	0.21	Protein intake	0.23	0.04	0.22
Model 5	CER	0.26	0.02	0.23	Protein intake	0.23	0.04	0.24

CER: creatinine excretion rate. Model 1 is unadjusted; Model 2 is adjusted for model 1 and age and gender; Model 3 is adjusted for model 2 and BMI and leg length; Model 4 is adjusted for model 3 and pack-years; Model 5 is adjusted for model 4 and eGFR < 60 mL/min/1.73 m^2^, polyneuropathy, and presence of macrovascular disease.

## Supplementary Materials

The following are available online at https://www.mdpi.com/2077-0383/9/10/3104/s1, Figure S1: flow chart of patient inclusion, Table S1: linear regression analyses for total steps per day.
