# Supplementary material for: Low Physical Activity in Patients with Complicated Type 2 Diabetes Mellitus Is Associated with Low Muscle Mass and Low Protein Intake
Source: J Clin Med. 2020 Sep 25;9(10):3104. doi: 10.3390/jcm9103104 (PMC7601707; doi:10.3390/jcm9103104)
Supplement: Supplementary file 1 [file jcm-09-03104-s001.pdf]

Journal of Clinical Medicine

MDPI

Supplementary files

Table 1. linear regression analyses for total steps per day.

Characteristics	Model 1	Model 2
	Standardized β	Standardized β
Age, years	−0.30 *	
Gender, % men	−0.04	
BMI, kg/m^2	−0.24 *	−0.25 *
Education level, (%)	0.15 *	0.13
Waist circumference, cm	−0.25 *	−0.24 *
Hip circumference, cm	−0.21 *	−0.22 *
Leg length, cm	0.24 *	0.21 *
Fat percentage, %	−0.18 *	−0.24 *
Predicted muscle mass, %	0.20 *	0.30 *
Urinary creatinine excretion, mmol/24h	0.26 *	0.22 *
Systolic blood pressure, mmHg	0.03	0.03
Diastolic blood pressure, mmHg	0.17 *	0.07
Pulse rate, bpm	−0.04	−0.08
Diabetes duration, years	−0.11 *	<0.01
Insulin use, yes (%)	−0.09	−0.02
Units of insuline	−0.15 *	−0.11
Alcohol intake	0.04	0.04
Pack years	−0.25 *	−0.25 *
Hba1c, mmol/mol	−0.04	−0.03
Total cholesterol, mmol/l	0.02	0.04
HDL-cholesterol, mmol/l	0.22 *	0.29 *
LDL-cholesterol, mmol/l)	0.01	0.03
Microvascular complications, %	−0.26 *	−0.17 *
Nephropathy, %	−0.25 *	−0.17 *
eGFR < 60 mL/min/1.73m^2	−0.30 *	−0.22 *
micro-albuminuria	−0.21 *	−0.16 *
Polyneuropathy, %	−0.23 *	−0.18 *
Retinopathy, %	−0.05	−0.01
Macrovascular complications, %	−0.21 *	−0.17 *
Coronary artery diseases, %	−0.19 *	−0.17 *
Cerebrovascular accident or transient ischemic attack, %	−0.21 *	−0.15 *
Peripheral arterial diseases, %	−0.09	−0.07
Amputation, %	−0.11	−0.11
Ureum excretion, mmol/24h	0.25 *	0.24 *
Protein intake, g/day	0.25 *	0.23 *
Protein intake, g/kg/day	0.31 *	0.32 *

Model 1: unadjusted. Model 2: adjusted for age and gender. * $p < 0.15$. BMI: Body Mass Index, eGFR: estimated glomerular filtration rate

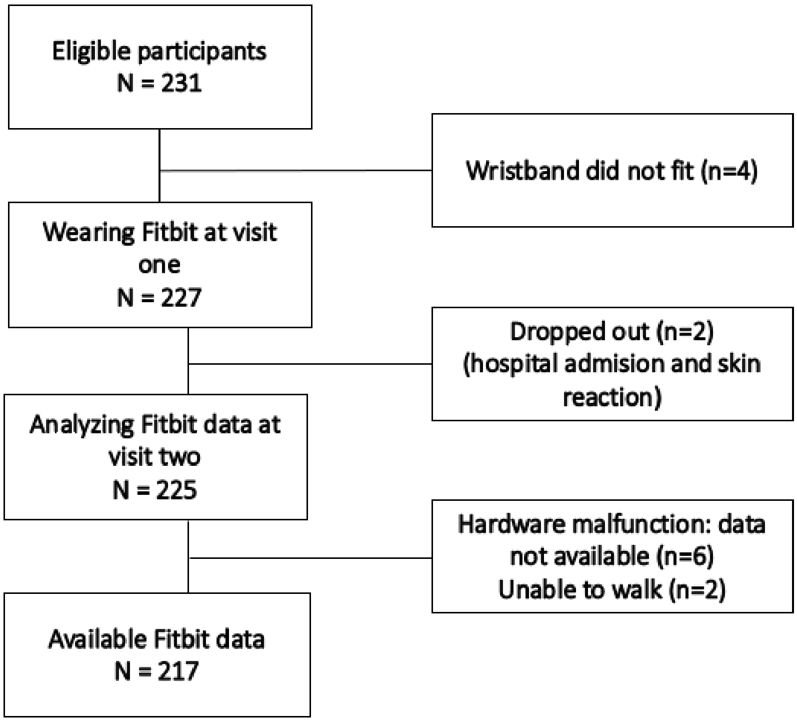

Figure 1. flow chart of patient inclusion.
